# Dental Education of the Public

**Published:** 1899-04

**Authors:** 


ARTICLE VI.

### DENTAL EDUCATION OF THE PUBLIC.

That a higher standard of both general and professional education than is now in vogue is needed for the advancement of the best interests of the dental profession, is a fact so patent and palpable as to require no further proof than has already been presented.

That much good will result both to the profession and public when a higher standard is fully established requires no argument, but no matter how well educated professionally, technically and generally the dentist may be, he can not reach the highest plane of his usefulness unless aided by an intelligent appreciation of his efforts on the part of his patients.

Generally speaking such appreciation is the exception rather than the rule, and this being the case, the cause is not difficult to find. The public are, as a rule, grossly ignorant of the value of, and necessity for, truly skillful dental services.

Undoubtedly the first serious dental lesion was toothache, and its first radical cure, extraction; and the world has not yet risen to a plane of intelligence where it demands positively an abatement of both these evils.

How the people at large are to be reached and a sufficient amount of dental knowledge imparted to them, are questions demanding our serious consideration.

The methods suggested include the earnest and oft repeated advice of the dentist to his parents at the chair, the instruction of the pupils in our public schools and the enlightenment of the general public through the columns of the daily press.

Of the first, let us say we believe it to be one of the fundamental duties of every dentist to give his patients at all times his most skillful services and the best advice of which he is capable.

But in this way but comparatively few can be reached; the method must be continued indefinitely, but it needs supplementary effort of some sort.

Our public schools probably offer us the best channel through which to disseminate dental knowledge to the people.

What children are told in school they usually accept as a fact; statements made by their teachers. particularly if oft repeated, have considerable weight and influence, and we believe this is the place where the most direct good can be done in educating the public to an appreciation of the value, care and preservation of their teeth. The instruction, however, should begin in our normal schools, and those who are to become teachers should receive adequate instruction in all that pertains to dentistry as the public should know it. This should be made an important part of their training and its value so emphasized as to leave a lasting and valuable impression. The text books used in our schools should contain also much more matter on this subject than they do at present, for many of them, so far as dental instruction is concerned, are now practically worthless, and every effort should be made to have this line of instruction at least clear, comprehensive and practical.

As for instructing the people through items published in the daily papers, we feel that there is so little to commend this method that it will hardly be attempted seriously by any one.

If our papers were clean and honest; if what they publish was reliable; if the people had faith in the statements printed; if the papers were not already overburdened with startling announcements of wonderful medical and dental discoveries and achievements, published at so much per inch by professional outcasts and charlatans; if reliable dental information could be published as coming from a reputable dental society and not from an individual, who, while hoping to educate the public a little, hopes more to fill his office with paying patients; if, we say, all these things could be, there might be some hope of using the newspapers in a clean, honest, legitimate way for the good of the public and the profession. But, under existing circumstances, we believe the less of this sort of public education is attempted the better it will be both for him who would attempt it and for the profession he should feel it an honor to represent.—EDITORIAL in *Pacific Medico-Dental Gazette.*

